# Immunological Mechanisms of Sickness Behavior in Viral Infection

**DOI:** 10.3390/v13112245

**Published:** 2021-11-08

**Authors:** Mia Krapić, Inga Kavazović, Felix M. Wensveen

**Affiliations:** Department of Histology and Embryology, Faculty of Medicine, University of Rijeka, 51000 Rijeka, Croatia; mia.krapic@uniri.hr (M.K.); ikavazovic@uniri.hr (I.K.)

**Keywords:** infection, sickness behavior, metabolism, appetite, cytokines, T cells, coronavirus, cytomegalovirus, anorexia, nausea, metabolic disease, diabetes

## Abstract

Sickness behavior is the common denominator for a plethora of changes in normal behavioral routines and systemic metabolism during an infection. Typical symptoms include temperature, muscle weakness, and loss of appetite. Whereas we experience these changes as a pathology, in fact they are a carefully orchestrated response mediated by the immune system. Its purpose is to optimize immune cell functionality against pathogens whilst minimizing viral replication in infected cells. Sickness behavior is controlled at several levels, most notably by the central nervous system, but also by other organs that mediate systemic homeostasis, such as the liver and adipose tissue. Nevertheless, the changes mediated by these organs are ultimately initiated by immune cells, usually through local or systemic secretion of cytokines. The nature of infection determines which cytokine profile is induced by immune cells and therefore which sickness behavior ensues. In context of infection, sickness behavior is typically beneficial. However, inappropriate activation of the immune system may induce adverse aspects of sickness behavior. For example, tissue stress caused by obesity may result in chronic activation of the immune system, leading to lasting changes in systemic metabolism. Concurrently, metabolic disease prevents induction of appropriate sickness behavior following viral infection, thus impairing the normal immune response. In this article, we will revisit recent literature that elucidates both the benefits and the negative aspects of sickness behavior in context of viral infection.

## 1. Introduction

Infection has a major impact on our systemic physiology. Especially when the pathogenic load is high, we display symptoms such as nausea, muscle weakness, tiredness, and temperature, which are generally referred to as sickness behavior [1]. Whereas we experience these changes as pathology, in fact, they are a carefully regulated response. Following infection, the immune system induces changes of systemic metabolism through the secretion of cytokines, which either alter metabolism of tissues directly or change normal endocrine control of systemic metabolism [2,3]. Concurrently, the immune system itself undergoes major changes in its requirement and consumption of nutrients. It has been estimated that during infection, the activated immune system may use up to 30% of all nutrients in the body [4]. Counterintuitively, despite its increased demand for nutrients, the activated immune system discourages nutrient intake whilst promoting metabolic changes that lower nutrient release in the bloodstream, thus restricting systemic nutrient availability [5]. The purpose of metabolic changes in response to infection appears to be twofold. On the one hand, the immune system restricts nutrient availability to most organs to limit pathogen replication. This is particularly true for glucose, which is a nutrient that many pathogens both of viral and bacterial origin use to promote their replication [6,7]. As a result, infection promotes a form of metabolism that is normally associated with fasting [5]. This is further enhanced by actual fasting due to reduced appetite and nausea. At the same time, the immune system aims to optimize access to nutrients by immune cells. Many activated immune cells, such as CD8 T cells and pro-inflammatory macrophages, favor glycolysis for their metabolic needs [8]. Therefore, these cells tend to highly upregulate glucose transporters on their cell surface, allowing them to take up enough glucose even when systemic concentrations are low. At the same time, many immune cells use fatty acids and ketones to fulfill their metabolic needs [9,10], allowing them to operate even in context of the fasting-type metabolism that is induced in response to infection. As such, both systemic metabolism and immunometabolism are adjusted in response to infection, and this is what we experience as sickness behavior.

The binal goals of immune-mediated changes to metabolism in response to infection can become pathological, most notably in context of metabolic syndrome (MS). MS is a condition that is typically the result of chronic obesity and is associated with increased blood pressure, high blood sugar, excess abdominal fat, hyperlipidemia, and increased blood cholesterol levels. When patients have at least three of these conditions, they are diagnosed with MS [11]. Immune-mediated changes to systemic- and immune-metabolism are negatively impacted by MS in two ways. On the one hand, because of deregulated systemic metabolism, people with MS cannot induce optimal metabolic changes to fight infection. People with MS therefore experience more infections, with longer duration and more severe symptoms [12]. For example, following infection with SARS-CoV-2 or influenza, people with diabetes mellitus type 2 (T2D) showed an increased risk of developing severe symptoms and had a higher mortality rate [13,14]. On the other hand, many immune-mediated changes of systemic metabolism are pro-diabetic. As a result, the immunological response to infection can aggravate symptoms of MS, such as defective blood glucose regulation [2,15].

In this review, we will revisit recent literature on the molecular mechanisms underlying the impact of the activated immune system on sickness behavior following infection. A particular focus will be on viral infection.

## 2. Immune-Mediated Sickness Behavior in Response to Infection

Upon infection, a carefully regulated cascade of events is initiated in which the immune system plays a central role. Infected tissues communicate the stress of pathogen invasion to the immune system within minutes to hours after pathogen encounter through the secretion of alarm molecules, such as type-I interferons, and by upregulating stress ligands on their cell surface [16]. These events recruit innate immune cells to the site of infection, which are further activated by recognition of pathogen-associated molecular patterns (PAMPs) on microbes or infected cells. Consequently, these cells start fighting the infection and secrete more specialized cytokines that help protect nascent cells from being infected and activate the adaptive immune system [17,18].

Sickness behavior presents in many ways, each regulated through a particular mechanism but typically initiated by cytokines. The central nervous system (CNS) plays a central role in the mediation of sickness behavior and many cytokines, most notably IFN-γ, TNF, IL-1β, and IL-6, therefore directly affect our brain. The production of these signaling molecules correlates with sickness behavior, such as fever, inability to focus, behavioral changes, and fatigue [17]. The hypothalamus is the part of the brain that is most closely associated with maintenance of systemic homeostasis. Many inflammatory signals therefore converge on this part of the CNS, which then mediates sickness behavior. Under stressful conditions, such as injury, inflammation, or infection, the hypothalamus causes an increase of the body’s temperature. Pyrogenic cytokines, including IL-1β, TNF, and IL-6, stimulate the endothelium of the brain to produce prostaglandins, most notably PGE2. These prostaglandins bind and activate the receptor EP3R on neurons, resulting in a central signal that promotes release of norepinephrine in the body [19]. This in turn leads to heat-promoting behavior, such as vasoconstriction and shivering, as well as changes in the metabolic rate of cells in the periphery, for instance, through induction of thermogenesis in brown adipose tissue [18,20,21,22]. Fever promotes the immune response against infection in several ways. Increased body temperature has been shown to promote migration and adhesion of lymphocytes to the draining lymph nodes in infection through the T-cell thermal sensory pathway [23]. NK cells show increased cytotoxic activity and increased migration to sites of inflammation under conditions of increased body temperature [20]. Finally, CD4 T cells modulate their immunological behavior under conditions of moderate fever by induction of GATA-3 and promoting the secretion of IFNγ, IL-4, and IL-13 [24].

Fatigue is a common sickness behavior following infection, which can persist for months even after the pathogen is cleared [25]. Many processes mediated by immune cell activation can lead to fatigue. Pro-inflammatory cytokines produced in infection can cause fatigue through modification of metabolic pathways through an endocrine loop, initiated in the hypothalamus and amplified by the pituitary gland. This promotes the release of corticotrophin-releasing hormone, cortisol, and adreno-corticotropic hormones and disrupts nutrient homeostasis in tissues such as liver, muscle, and adipose tissue. Intravenous injection of TNF, IL-1β, and IL-6 was therefore shown to induce fatigue in humans [26], whereas inhibition of TNF signaling prevents this symptom [18]. Other causes of fatigue include oxidative stress induction and impairment of systemic metabolism [26]. Cytokines such as type-I interferons can directly stimulate the CNS and mediate fatigue through neuropsychiatric effects [27]. In addition, cytokines such as IFNγ promote insulin resistance (IR) in many tissues, including the muscle, thus reducing its glucose uptake and anabolic activity [2]. In addition, IR is associated with a shift in serum lipid concentrations, and people with acute infection have been shown to have an increase in free fatty acids (FFA) and triglycerides in circulation [28,29]. As a result, activity of glucose- intensive organs, such as skeletal muscle, is reduced, thus contributing to fatigue.

Another frequent manifestation of sickness behavior is anorexia, i.e., the loss or lack of appetite and subsequent weight loss. During infection, our immune system requires nutrients for energy and activation, but surprisingly, we reduce our food intake when we feel sick. Systemic administration of cytokines, such as TNF and IL-18, were shown to cause anorexia by directly targeting receptors in the central nervous system, most notably in the Stria Terminalis [30,31]. As a result, instead of using external sources of energy, sickness promotes the utilization of alternative, internal nutrient sources. For example, FFA are released from adipose tissue and are used as a source of energy in the liver, which cfan lead to weight loss [18]. The pro-anorexic effects of cytokines are not restricted to the CNS. Cytokines can act directly on adipose tissue and stimulate their production of hunger-suppressing adipokines, such as leptin. In hamsters, TNF and IL-1β produced in response to LPS administration were shown to increase leptin expression in adipose tissue and induce anorexia [32]. Leptin acts on the brain, where it inhibits feeling of hunger and lipogenesis in adipose tissue. Leptin is also important for the activation and proliferation of NK cells, T cells, and dendritic cells, suggesting that the benefits of its increased concentrations in the blood reach beyond control of food intake alone [5]. Finally, the pro-inflammatory cytokine TNF can directly trigger the expression of hormone-sensitive lipase in adipocytes. This enzyme hydrolyzes triglycerides, resulting in the release of FFA and triglycerides from adipose tissue. TNF administration can therefore cause a reduction of overall adipose tissue size and a concomitant increase of these molecules in circulation [18].

**Figure 1:**
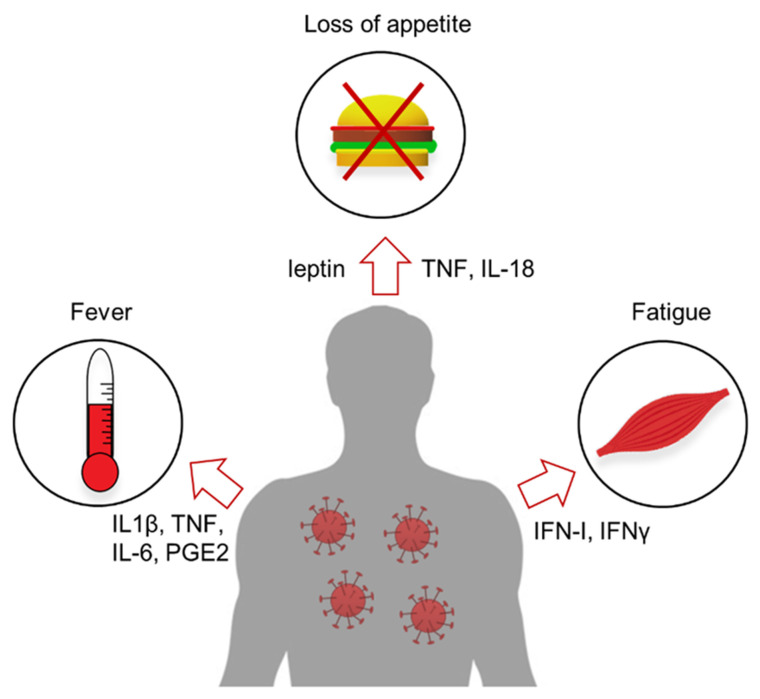
Immunological mediators produced in context of viral infection induce sickness behavior by targeting various organs in our body. Prime examples are (1) the induction of fever by cytokines. TNF and IL-6, which stimulate endothelium in the brain and promote their release of prostaglandin PGE2, which directly target the thermoregulatory center in the CNS; (2) induction of anorexia by cytokines such as TNF and IL-18, which promote release of leptin and suppress appetite; and (3) induction of fatigue by Type-I interferons and IFNγ, which directly target muscle cells.

In summary, in response to infection, a complex interaction between the immune, endocrine, and CNS transiently alters the systemic nutrient availability and consumption of peripheral organs. As a result, these tissues alter their metabolic state, which leads to sickness behavior (Figure 1).

## 3. Molecular Mechanisms of Immune-Mediated Changes in Metabolism following Infection

Whereas the impact of individual cytokines on systemic metabolism is becoming increasingly clear, how they mediate these effects on a molecular level has long remained uncertain. However, much progress has been made in recent years, especially for adipose tissue and the liver [33]. Adipose tissue is the body’s main storage location of excess nutrients in the form of triglycerides. Much study has been devoted to the impact of cytokines on adipose tissue in context of obesity, as this organ was shown to be the source of inflammatory processes that contribute to development of metabolic disorders [34]. IFNγ is one of the most highly induced cytokines in context of viral infection and has been shown to down-regulate PPARγ in adipocytes [35]. PPARγ is a master regulator of adipose differentiation, lipogenesis, and glucose metabolism. The murine 3T3-L1 cell line is commonly used to study adipogenesis, as these cells can be differentiated into mature adipocytes in vitro. When treated with IFNγ, IFN-β, or TNF, 3T3-L1 cells showed increased insulin resistance through reduction of PPARγ levels [36,37]. In human adipocytes, IFNγ-mediated PPARγ downregulation was shown to lead to a reduction in insulin-mediated glucose uptake, reduced surface expression of the glucose transporter GLUT4, and therefore to decreased triglyceride storage [38]. Similarly, IL-1β reduces insulin-mediated glucose uptake and expression of the GLUT4 glucose transporter in adipose tissue [39]. The impact of typical Type-2 cytokines, such as IL-4, on adipose cell biology is much less clear. Whereas treatment with IL-4 reduced adipogenesis and increased lipolysis in adipose 3T3-L1 cells, in primary rat adipocytes this cytokine prevented lipolysis [40,41]. Cytokine-mediated lipolysis can become pathological if it induces a complete breakdown of adipose and muscle tissue, a state that is referred to as cachexia. Cachexia is a common phenomenon in late-stage cancer patients but can also occur in context of severe or chronic infection [42]. In a mouse model of chronic lymphocytic choriomeningitis virus (LCMV) infection, serum levels of triglycerides and FFA were strongly increased because of excessive lipid secretion and reduced lipid uptake by adipose tissue, resulting in cachexia. Chronic LCMV infection caused reduction in mRNA expression of the DGAT2 and LPL enzymes involved in triglyceride synthesis and hydrolysis of triglycerides from lipoproteins, respectively. These changes were associated with IFN-I-mediated stimulation of CD8 T cells. How they lead to a “point of no return” in lipid degradation still requires investigation [42].

The liver is one of the primary organs in the body responsible for maintenance of systemic homeostasis of nutrients, such as glucose, vitamins, and lipids. In that role, the liver is involved in a plethora of metabolic processes, including the processing and synthesis of carbohydrates, proteins, and fatty acids [43]. Not surprisingly, the liver also plays a key role in mediating systemic metabolic changes in context of infection. Cytokines such as IL-1β and IL-6, molecules for which expression is highly induced following viral infection, bind to their receptors on hepatocytes [44]. In response to these cytokines, hepatocytes upregulate expression of complement factors as well as antimicrobial proteins and peptides, such as hepcidin [45]. For example, acute influenza virus infection was shown to increase the expression of hepatic enzymes involved in metabolism of fatty acids, leading to lipid accumulation in liver cells [46]. In addition to being a metabolic organ, the liver is also an endocrine gland that impacts regulation of systemic metabolism through the secretion of hormones. Hepatocytes produce insulin-like growth factor (IGF-1), which is an anabolic hormone for which expression is strongly increased in response to infection. Moreover, in mice, IGF-1 was shown to enhance weight loss following influenza infection [47].

Typically, infection-induced changes of systemic metabolism are of transient nature, but in case of chronic infection of the liver, for example, with the hepatitis C virus (HCV), long-lasting changes and tissue damage may be induced. In chronic HCV infection, hepatocytes were shown to have impaired lipoprotein production, which could lead to dyslipidemia. Moreover, HCV infection led to increased expression of ferritin, resulting in the accumulation of iron and ultimately leading to liver toxicity [48,49,50].

Thus, both through direct and indirect actions, cytokines have a profound effect on systemic metabolism and the development of sickness behavior.

## 4. Nutrient Requirements of Activated Immune Cells

Following activation, immune cells undergo extensive changes in their metabolism to allow for rapid proliferation and acquisition of effector functions. Many excellent reviews on immunometabolism have been written [4,8], and we will therefore only briefly address this topic here. However, since the metabolism of immune cells is directly linked to their functionality, it is important to realize that the state of systemic metabolism therefore also impacts the efficiency of the immune system.

In absence of infection, resting immune cells, such as memory and naïve T cells, M2 macrophages, and dendritic cells, depend mostly on oxidative phosphorylation to fulfill their energetic needs, which is the most efficient way to make use of available nutrients for the generation of ATP [33,51,52]. When these cells become activated, their metabolic requirements shift, and they start using predominantly aerobic glycolysis to fulfill their energetic needs. Moreover, nutrients are shuttled into the pentose phosphate pathway to generate proteins, nucleic acids, and other building blocks that they need for their activation, proliferation, and growth [9,53]. Activated immune cells massively increase their consumption of high-energetic nutrients, such as glucose and glutamate. Following infection, therefore, up to 30% of all nutrients in the body can be directed towards the activated immune system [54,55]. As a result, in the absence glutamate, activated T cells have a strongly reduced ability to proliferate and produce cytokines [56]. Similarly, depletion of glucose inhibits production of IFN-γ, perforin, granzyme, and proliferation by activated T cells and NK cells [57].

Whereas infection causes an increase in nutrient requirements by the immune system, at the same time, the immune system reduces nutrient intake by inducing sickness behavior. This apparent paradox is resolved by a switch of systemic metabolism to a state normally associated with fasting. This form of metabolism depends on the utilization of triglyceride stores from adipose tissue and is characterized by an increased concentration of FFA and ketones in circulation [42]. At the same time, immune cells remain functioning optimally despite their increased needs of nutrients, such as glucose. One explanation is that activated immune cells strongly upregulate glucose transporters on their cell surface, allowing them to retain functionality even when glucose levels are as low as 0.5 mM [58]. Second, it appears that not all activated immune cells preferentially use glucose as a source of nutrients. B lymphocytes are essential for the generation of a high-affinity neutralizing antibody response following infection. They accomplish this after formation of a specialized structure called the germinal center, where activated B cells proliferate, differentiate, and undergo somatic hypermutation [59]. Surprisingly, B cells increase oxidative phosphorylation in the tricarboxylic acid cycle and not glycolysis early after activation [60]. Moreover, germinal center B cells predominantly use fatty acid oxidation as a primary energy source both in vitro and in vivo. Glycolysis is used minimally to fulfil metabolic needs by these cells [10]. Importantly, inhibition of fatty acid oxidation results in a strong reduction of germinal center formation in vivo [10]. Thus, the metabolism of various activated immune cells is in fact promoted by sickness behavior that mediates a switch to fasting-type metabolism.

Lipids are also important for the functionality of other immune cells. Macrophages play an important role in the early defense against pathogens. They require metabolic reprogramming to become activated, after which they produce pro-inflammatory cytokines and conduct their phagocytic activity. M1-activated macrophages were shown to increase lipid production through the mTOR signaling pathway, which increases their plasma membrane fluidity and facilitates phagocytosis of infected cells. Lipids can also increase phosphatidylcholine production, which activates the NLRP3 inflammasome and production of IL-1β and IL-18 by macrophages [61]. Cytokines themselves also play a major role in guiding the metabolic transformation of activated immune cells [62]. For example, IL-7, a cytokine important for lymphocyte development and growth, was shown to increase glucose uptake and glycolysis in T cells [63]. In contrast, IL-10, a molecule which inhibits immune cell activation and dampens the inflammatory response, impairs glycolysis and was found to inhibit maturation of activated dendritic cells [64].

**Figure 2:**
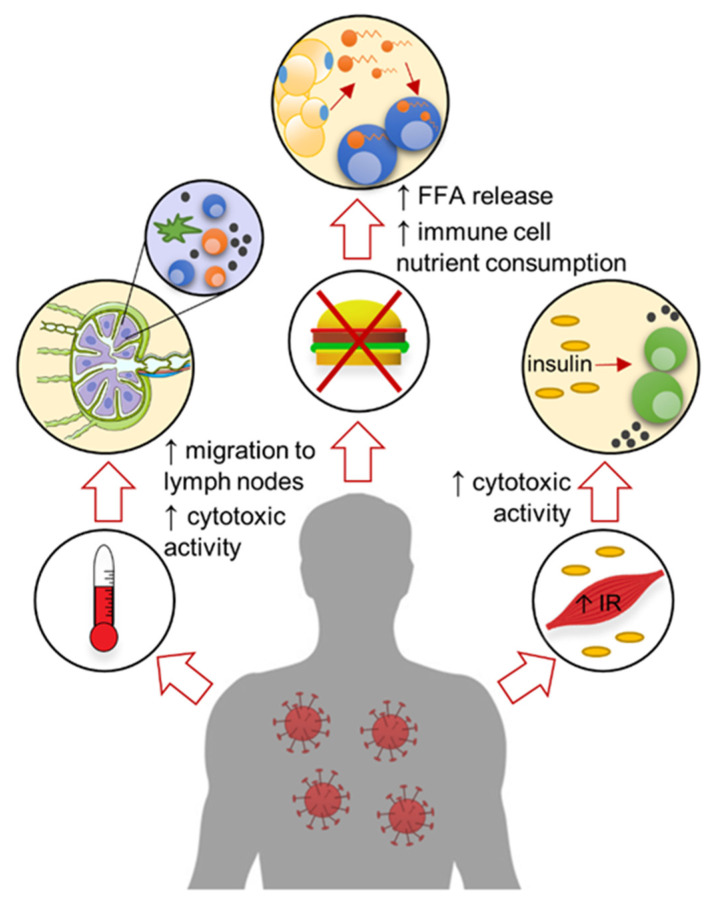
Sickness behavior is beneficial for immune cell activation. Fever can induce migration of cells to the lymph nodes and increase their cytotoxic activity. Loss of appetite induces the release of circulating free fatty acids (FFA) that are used as an energy source in immune cells. Muscle fatigue can be the consequence of insulin resistance (IR) and reduced glucose uptake, which increases systemic insulin levels and promotes the anti-viral T-cell response.

In summary, an important role of systemic metabolic changes manifested as sickness behavior is to facilitate immune cell metabolic reprogramming and activation to improve viral clearance (Figure 2).

## 5. Pathogen-Mediated Changes of Metabolism

Whereas the immune system mediates changes in systemic homeostasis to benefit the immune response, pathogens also induce metabolic changes in their host to promote their own replication. These changes are most profound in the cells that have been directly infected, yet indirectly, they can also have a major impact on metabolite levels in entire tissues and even in circulation. At a cellular level, these changes have been characterized most extensively for viral infection. Upon entry into the cell, a virus rapidly alters the metabolism of its host to optimize production of new viral particles [65]. The nature of these changes varies greatly between pathogens [66] but typically increases the metabolic rate of the cell in order to accommodate the increased demand of anabolic processes required for viral particle assembly. A primary target of viral reprogramming is carbon metabolism. Viruses such as human cytomegalovirus (hCMV), Epstein Barr virus, influenza A, and dengue virus upregulate glucose transporters at the cell surface to boost glucose uptake and increase the rate of glycolysis in the cell [6]. Medium containing low glucose levels therefore strongly impairs replication of viruses, such as hCMV, in cell culture. Viruses also increase the efficiency of glucose utilization. hCMV, herpes simplex virus-1 (HSV-1), and zikavirus all increase oxidative phosphorylation to optimize ATP production [6,67].

Apart from glucose, viruses also promote the utilization of other molecules, such as glutamine, as an energy source. Infection with vaccinia virus, HSV-1, and adenoviruses was shown to increase glutamate uptake and enhance the activity of glutaminase and glutamate dehydrogenase [6,67,68]. Moreover, α-ketoglutarate, the product of glutaminolysis, was able to maintain viral growth and energy production under conditions of limiting glutamate availability [6,67,69]. Finally, many viruses boost lipid synthesis in the cell to promote the formation of the viral envelope. Lipids enable many viruses to infect their host by serving as receptors or co-factors for the formation of endosomes, which is usually the first step of cellular entry [66]. For hCMV and influenza A, it was shown that viral replication is reduced upon the pharmacological inhibition of acetyl-CoA carboxylase (ACC) and fatty acid synthase (FAS) [70]. Thus, viruses can hijack several different metabolic pathways in order to boost cellular energy production and promote viral replication.

In addition to direct effects of the virus on the metabolism of the cells it has entered, infection may indirectly impact the metabolic state of the entire tissue in which they reside. Metabolic changes in infected cells alter the tissue-microenvironment of an organ and thereby its consumption of nutrients. This results in an altered endocrine signal coming from these organs, which causes a systemic response to restore homeostasis. For example, the liver is an important metabolic organ responsible for maintaining homeostasis of many different factors in circulation including lipids, glucose, and vitamins. Following LCMV infection of hepatocytes, the urea cycle becomes dysregulated, causing a relative decrease in ornithine in favor of arginine [71]. This shift in systemic nutrient availability has a direct negative impact on the ability of the body to fight the infection. In-vitro treatment of CD8 T cells with arginine resulted in a poor anti-viral response. Notably, arginase treatment of mice caused a reduction in TNF and IFN-γ production by CD8 T cells, which was associated with aggravated liver damage. Thus, the impact of viruses on hepatic urea metabolism appears a targeted strategy to impair the local immune response [71].

Viral infection was also shown to have a profound impact on systemic lipid levels. LCMV infection was shown to increase lipids in circulation. Trem2 is a protein that is important for lipid sensing. Animals with specific deficiency of Trem2 on myeloid cells therefore showed reduced viral titers and had less liver pathology upon LCMV infection [72]. Influenza A was shown to target the ability of the body to cope with oxygen radicals. Early after infection of mice with this virus, a reduction was observed in the levels of antioxidants, such as glutathione, in the liver [73]. Glutathione is one of the most potent antioxidants in our body and has been shown to be important for the response to viral infection [74]. Viruses, such as dengue, can target hepatocytes and cause liver damage by the induction of oxidative stress. Reactive aldehyde malondialdehyde levels and the ratio of GSSG and GSH were increased in infection, whereas superoxide dismutase was decreased, indicating increased sensitivity to oxygen radicals. Exogenous glutathione treatment impaired the reactive oxygen species production and thereby reduced viral entry into cells, thus illustrating the detrimental effect of oxygen radicals in context of infection [75].

Thus, through the induction of local changes in the cells that they infect, viruses can have a major impact on systemic metabolism, which impairs the antiviral immune response and promotes their replication.

## 6. Detrimental Effects of Metabolic Disease on Sickness Behavior

Patients with metabolic syndrome are well known to acquire common infections more frequently, and typically, the duration of disease is longer and with an increased risk of complications [76]. This has been best documented for patients with T2D, a disease in which people fail to maintain blood glucose levels below certain threshold values. People with T2D were shown to have more respiratory infections, urinary tract infections, and bacterial infections and showed a significant increase of infection-related death [14,77,78,79]. More recently, T2D was shown to significantly increase the risk of developing severe complications and death because of SARS-CoV-2 infection [13,80].

Various underlying causes have been proposed for the increased susceptibility of patients with metabolic syndrome to infection. These include reduced access of immune cells to infected tissues due to microvascular damage [81], disruptive immune cell skewing due to adipose tissue inflammation [3,34], and changes in endocrine hormones that can directly affect immune cell function [82,83]. However, aberrant sickness behavior appears to be a more prominent cause of immune disfunction in context of metabolic syndrome. As mentioned, a key sickness behavior is reduced nutrient intake and a shift to fasting metabolism, characterized by lower blood glucose levels [30]. Especially people with T2D have chronic hyperglycemia, meaning that they fail to lower blood glucose levels following infection. Hyperglycemia was shown to have a direct, detrimental effect on immune cell function [84,85]. Indeed, the level of glycated hemoglobin (HbA1c) positively correlates with the duration and severity of infection with a number of viral and bacterial pathogens [84]. Experimental hyperglycemia induced in mice by streptozotocin injections directly impairs macrophage activation in response to infection with *Mycobacterium tuberculosis* (TB). As a result, neutrophil recruitment and dendritic cell activation are reduced, causing a decrease in the cytokine production by these cells [86]. In addition, CD4 and CD8 T cells as well as NK cells showed reduced cytotoxicity and cytokine production in T2D patients infected with TB as a direct result of hyperglycemia [86].

Apart from providing an optimal metabolic environment for immune cells, a key purpose of inducing fasting metabolism in response to infection is to restrict nutrient availability to pathogens. Indeed, anorexia promotes the response to viral infection, which is counteracted by hyperglycemia [87]. At a molecular level, nutrient restriction promotes the innate stress response of cells to infection. Following a meal, muscle cells quickly take up glucose that enters the blood stream to lower glycemia, but a large fraction is returned into circulation in the form of lactate [88]. This lactate is used by many tissues as their primary source of energy [88]. In addition, lactate was shown to have an anti-inflammatory role, as it inhibits RIG-I signaling in response to viral RNA detection and impairs the subsequent type-I interferon response [89]. People with MS typically have increased levels of lactate in circulation [90], which impairs the innate response to viral infection.

Thus, metabolic syndrome negatively impacts the normal immune response to viral infection by preventing the induction of a metabolic state that is optimal for immune cell function and limits pathogen replication.

## 7. Detrimental Effects of Sickness Behavior on Metabolic Disease

Due to the close interaction between the immune system and tissues involved in regulating systemic metabolism, it is not surprising that infection also negatively impacts metabolic disease. Among endocrinologists, it is well known that infection causes major changes in blood values of key metabolic parameters, such as glucose, in patients with T2D. International guidelines therefore recommend screening for infection in patients that newly present with metabolic diseases, such as T2D [91]. However, the underlying molecular mechanisms only now start to become apparent. Pro-inflammatory cytokines, such as TNF and IFNγ, have long been known to induce systemic IR and patients with T2D have indeed chronic systemic presence of low levels of these cytokines [92]. Adipose tissue in obese individuals accumulates pro-inflammatory immune cells that respond to metabolic stress of hypertrophic adipocytes, resulting in the release of cytokines in the bloodstream [3,34]. However, barring severe infections, such as SARS-CoV-2 [15], most viruses do not typically induce an increase in blood glucose levels [2]. The increase of pro-inflammatory cytokines does lead to insulin resistance, most notably in skeletal muscle. This is normally compensated by increased insulin production of the pancreas [58,93,94], but it long remained unclear what the physiological benefit of this effect was to the host.

Insulin is an anabolic hormone that shares many of its downstream signaling components with those of co-stimulatory molecules, such as CD28 [95]. These two types of receptors therefore share several functional properties, such as the upregulation of glucose transporters on the cell surface upon ligand binding [95,96]. Many immune cells, including effector CD8 T cells, express the insulin receptor on their cell surface and can be stimulated by this hormone to increase their effector function (Figure 2 [61,97]). A key goal of infection-induced insulin resistance is therefore to increase systemic insulin levels in order to promote the effector CD8 T-cell response [2]. The sickness behavior associated with this phenomenon is that muscle cells take up nutrients with lower efficiency, thus contributing to muscle weakness. In metabolic disease, many people have underlying IR, which in some, has progressed to such a degree that blood glucose levels are beyond certain well-defined threshold levels, leading to the diagnosis of T2D. If people have increased blood glucose levels that do not yet reach these threshold levels, we speak of pre-diabetes, which is a high-risk state for development of T2D [98]. If metabolic changes induced by infection come on top of pre-existing IR, it may lead to progression from pre-diabetes to diabetes or worsen the current metabolic state of people with T2D [2,15]. Indeed, infection with several pathogens, such as hCMV, HCV, and SARS-CoV-2, were shown to be a risk factor for the development of T2D [15,99,100].

Apart from immune-mediated changes to systemic insulin levels, pathogen-induced alterations of insulin secretion have also been documented. Many pathogens, most notably viruses, can infect the pancreas and negatively impact insulin output by this organ. Indeed, several viruses have been associated with the destruction of pancreatic β-cells and the induction of type 1 diabetes [101]. Recently SARS-CoV-2, the virus that causes COVID-19, has been shown to infect and replicate in cells of both the exocrine and endocrine pancreas [102]. However, these observations were made on material obtained post-mortem. In addition, SARS-CoV-2 was shown to drive increased insulin output by the pancreas [15]. Whereas this pathogen therefore has a long-term detrimental effect on pancreatic β-cell numbers, its insulin output is currently unclear.

In summary, the metabolic changes induced by viral infection in order to mediate sickness behavior overlap to a certain extent with those observed in context of metabolic syndrome. Notably, it seems that some aspects of MS are in fact derailed sickness behavior reserved for the normal physiological response against viral infection. If MS and viral infection therefore co-occur in the same individual, it may lead to an aggravation of metabolic disease.

## 8. Conclusions

Sickness behavior in response to viral infection is not a pathology but a carefully orchestrated response mediated by the immune system. Its purpose is to optimize the immune response against the invading pathogen whilst minimizing viral replication in infected cells. The cytokine profile that is induced in response to a specific infection determines the metabolic change that is accomplished and thereby the nature of the sickness behavior. Sickness behavior can become pathological, for example when lipid degradation aggravates to cachexia in context of severe infection. In addition, inappropriate sickness behavior, such as the inflammatory response occurring in obesity, may contribute to the formation of metabolic disease. At the same time, metabolic disease prevents the development of appropriate sickness behavior following viral infection, thus impairing the immune response and increasing susceptibility to infection. In summary, immunology and metabolism are tightly interdependent in the fight against infection both at a cellular and a systemic level. Further research in this field will therefore benefit treatment of both metabolic and infectious disease.

## Data Availability

Not applicable.
